# When Connectedness Increases Hemispatial Neglect

**DOI:** 10.1371/journal.pone.0024760

**Published:** 2011-09-27

**Authors:** Yanghua Tian, Yan Huang, Ke Zhou, Glyn W. Humphreys, M. Jane Riddoch, Kai Wang

**Affiliations:** 1 Department of Neurology, the First Hospital of Anhui Medical University, Hefei, Anhui Province, People's Republic of China; 2 State Key Laboratory of Brain and Cognitive Science, Institute of Biophysics, Chinese Academy of Sciences, Beijing, China; 3 Graduate University, Chinese Academy of Sciences, Beijing, China; 4 Behavioural Brain Sciences, School of Psychology, University of Birmingham, Birmingham, United Kingdom; University of Minnesota, United States of America

## Abstract

Patients with left neglect were tested with “chimeric” figures composed of the right and left halves of two different objects. The connectivity relation was modulated between the two half figures. For some displays, the two chimeric halves were separated by a small gap, while in others, the separate halves were connected by a line segment. In line with previous reports, performance on reporting the left half improved when the chimera were separated; but when a line connected the two separated halves the advantage was lost. If the connecting line was broken, the performance was again enhanced. The results suggest an important role for connectedness in the representation of perceptual objects and in the distribution of attention in neglect.

## Introduction

Patients with right hemisphere lesions [1], especially of the right inferior parietal lobe [2] or right superior temporal cortex [3] often fail to be aware of stimuli and events which fall toward the contralesional side. Neglect is considered as a lateralized impairment in the distribution and orienting of visual attention [4]–[5]. Investigations on neglect can provide insights into the mechanism of attentional selection. The issue whether neglect operates in space- or object-based coordinates (i.e., egocentric and allocentric neglect) has been controversial ([e.g.], [ 6]–[13]). Driver and Halligan were among the first researchers to demonstrate how neglect of the left-side of an object remained even when the object was tilted so that the left side of the object fell on the right side of space [8]. On the basis of this finding, they suggested that neglect can occur in both allocentric and egocentric co-ordinates. Somewhat more recently, Driver and Pouget [14] proposed that such effects can still be accounted for in egocentric terms if the object is considered in relation to its position on the retina and there is a retinally graded deficit in neglect. They state that “relative retinal position can matter as much as absolute retinal location” with detriment of visual attention to left-side objects, or the left side of objects. However, retinal effects cannot be used to account for those patients who demonstrate ‘paradoxical neglect’ where neglect is for the left for single perceptual objects (e.g., there may be errors on the left when reading words, within-object neglect) but for the right when there are multiple perceptual objects (e.g., when reading the letters in a word, there are omissions of the right-side letters – between-object neglect). This form of neglect is relatively rare and has been observed both after unilateral left [15]–[17] and bilateral parietal lesions [18]. Data such as these have led to the proposal that within- and between-object spatial relations are coded separately and in parallel [17]–[19]. Recently Chechlacz and colleagues [20] have reported that there were separate anatomical substrates for these two forms of neglect.

The distinction, between within- and between-object spatial codes, can be used to account for the pattern of neglect reported by Young, Hellawell and Welch [13] and Buxbaum and Coslett [21]. In these studies, poor performance in reporting the left half of chimeric figures was improved when a gap was introduced between the two chimeric halves. In these instances, patients showed left allocentric neglect when parts configured to form faces but not when they did not (space-based processing was unimpaired), and this occurred irrespective of the positions of the parts in the field. Interestingly, Young and co-workers' patient did not show left neglect when presented with two left sides of faces placed alongside one another. Young et al. suggested that neglect was apparent only when the parts of the object (the two halves of the face) configured to form a “good” perceptual object (e.g. having closure). These findings stand in contrast to studies of patients with visual extinction where performance is usually good when the patients have to report single stimuli whether they are presented in the contra- or ipsi-lesional field. If two items are presented simultaneously (i.e., under conditions of competition for attentional resources), patients typically fail to report the contralesional item. However, if the two items group in some way so they become a single perceptual unit rather than multiple units, performance improves [22]. Low level grouping cues such as collinearity [23]–[24], connectedness [25]–[26], common shape [23], [25], common contrast polarity [23], [25], and common region [25] have all been shown to be effective. The seeming paradox between the results from the two groups of studies may be resolved by considering the differences in stimuli. Higher order stimuli are used in the Young et al. [13] and Buxbaum and Coslett [21] studies (e.g., faces and line drawings of animals) compared with simple geometric shapes in the other studies. Chimeric objects are visually complex relative to the geometric shapes, a factor which is known to influence ipsilesional disengagement of attention from the ipsilesional side [27]. In addition, the meaningfulness of ipsilesional information may also affect attentional processing. Seron, Coyette and Bruyer [28] showed better performance (reduced neglect) if picture stimuli could be identified from feature information that was present on the right side (e.g., a rhino facing to the right may be identified by its horn, but when facing the left may be misidentified as ‘animal’ or ‘hippopotamus’) (see also [18]). Visual complexity and stimulus meaningfulness may underlie the neglect of the chimeric stimuli used in the Young et al. and the Buxbaum and Coslett studies.

The present study sought to investigate the influence of a perceptual object based on connectedness on neglect. Do the beneficial effects of connectedness with low-level (meaningless) stimuli (e.g., geometric shapes) also hold with higher-level (meaningful) objects? Humphreys and Riddoch [29] showed beneficial effects on contralesional stimulus report by joining simple geometric shapes (circles) with a line. Will similar effects hold with more visually complex stimuli? For instance, will two half-figures belonging to different objects be perceived as a single perceptual unit when they are connected by a line and allow identification of the contralesional chimeric or will stimulus complexity and meaningfulness result in allocentric neglect when chimeric halves are connected?

Here we report two experiments. In Experiment 1 we manipulated connectedness by either having a line or a gap between two separate half-figures, and performance in these conditions was compared with performance with the original chimeric figures. In Experiment 2 we sought to generalize our findings by using a random curve to represent general connectedness and a broken line to represent the gap condition rather than merely spatial isolation. We examined whether connecting the two halves alleviated or aggravated spatial neglect.

## Methods

### Patients

**Figure 1:**
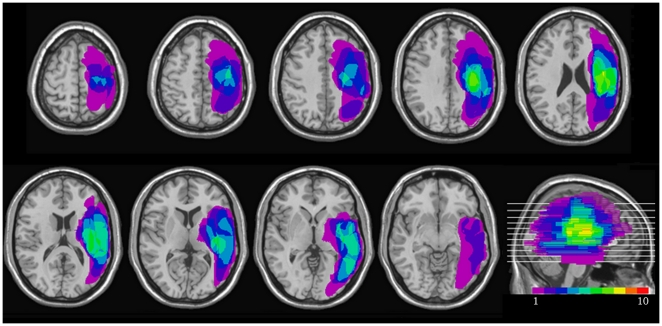
Lesion overlap after summation for all neglect patients (n = 10).

Ten patients (nine males, one female) with left unilateral neglect participated (10 in Experiment 1 and 9 in Experiment 2) and provided written consent. Patients were all right handed and had normal or corrected-to-normal vision. Detailed information of each patient is described in Table 1. They suffered from ischemia, or haemorrhage, but did not suffer from any additional impairment such as as on-going neurological diseases, psychiatric disorders, or dementia. The assessments and experiments were conducted at least two weeks after their stroke or brain trauma. An initial series of diagnostic tests were performed to assess visuospatial neglect: the Albert cancellation task [30], a line bisection test [31], copying a daisy [32], and Star Cancellation test [33]–[34] (see Table 1). The sites of the lesions were documented by means of CT or MRI scans (see Fig. 1).

**Table 1:** Demographic and clinical characteristics of hemispatial neglect patients.

Patient	Sex	Age	Edu	Time	Etiology	ALB	LB (%)	SC	Copy	VFD	Lesion
**ZGS**	**M**	**70**	**14**	**400**	**H**	**L:9/18 R:12/18**	**44**	**L:11/27 R:23/27**	**2**	**Yes**	**T-P**
**QJB**	**M**	**51**	**15**	**14**	**H**	**L:14/18 R:18/18**	**35**	**L:18/27 R:27/27**	**1**	**Yes**	**BG-T**
**ZMY**	**F**	**68**	**8**	**3**	**I**	**L:11/18 R:18/18**	**46**	**L:17/27 R:27/27**	**2**	**No**	**T-P**
**RL**	**M**	**58**	**8**	**2**	**I**	**L:2/18 R:15/18**	**61**	**L:4/27 R:18/27**	**2**	**No**	**T-P-O**
**RMX**	**M**	**63**	**10**	**16**	T	**L:0/18 R:17/18**	**70**	**L:7/27 R:20/27**	**1**	**No**	**F-T**
**HM**	**M**	**44**	**11**	**3**	**I**	**L:5/18 R:15/18**	**13**	**L:4/27 R:26/27**	**2**	**No**	**T-P-O**
**ZDG**	**M**	**57**	**3**	**6**	**I**	**L:12/18 R:18/18**	**41**	**L:14/27 R:26/27**	**4**	**No**	**T-P-O**
**WJW**	**M**	**46**	**5**	**24**	**H**	**L:9/18 R:16/18**	**74**	**L:12/27 R:23/27**	**4**	**No**	**F-T-P**
**RHY**	**M**	**56**	**14**	**6**	I	**L:0/18 R:8/18**	**16**	**L:0/27 R:7/27**	**1**	**No**	**F-T-P**
**TDF**	**M**	**58**	**5**	**2**	**I**	**L:8/18 R:14/18**	**57**	**L:16/27 R:25/27**	**1**	**No**	**T-P**

Note: Edu-education (years); Time-weeks post the onset of stroke or brain trauma at time of testing; I-ischemic; H-haemorrhage; T-trauma; ALB-Albert' test ; LB-Line bisection (% deviation for line); SC-Star Cancellation test;Copy-copying a daisy; VFD-visual field defect; F-frontal; T-temporal; P-parietal; O-occipital; BG-basal ganglia.

### Apparatus

The stimuli were presented at the center of a 14.1-inch HP laptop computer set at 60 Hz, which was laid on the right side of the patients' body midline at a viewing distance of approximately 60 cm. The figures were dark against the light screen. Eye movements were neither limited, nor monitored.

### Stimuli

The chimeric figures in this study were generated from 30 drawings of animals and common objects from Snodgrass and Vanderwart [35]. Prior to the experimental studies each patient's ability to recognize all the pictures was assessed. These pictures could be recognized by all patients. Three types of stimuli (Chimeric, Chimeric-Gap, Chimeric-Connected) were tested. The Chimeric figures were generated by conjoining right halves of these figures with non-matching left halves. The Chimeric-Gap figures were created by introducing a 3-cm gap between the halves of the Chimeric figures (Exp. 1) or adding a broken line (i.e., two 1.5° parallel lines separated 1° vertically) in the gap (Exp. 2). And the last type, Chimeric-Connected figures were created by connecting the separated chimeric halves by a 3° long and 4.5 point wide line (Exp. 1) or a random curve with the same width (Exp. 2). The Chimeric figures ranged in size from 8°×8° to 14°×6°; the Chimeric-Gap and Chimeric-Connected figures were the same height but 3° wider horizontally. There were 20 figures for each condition, thus a total of 60 figures were presented in each experiment. The same chimerics appeared in each of the three chimeric conditions. Except for the connecting line, the same stimuli were used in Experiment 1 and 2.

### Procedure

The procedure of the experiment was almost the same as that employed by Buxbaum and Coslett [21]. They were asked to name the figures without being told that the figures were in any way unusual. Only a single prompt (“Anything else?”) was given when they missed some part of the stimuli. All the 60 figures were presented once in a random order. In an effort to eliminate floor and ceiling effects, a preliminary experiment was conducted to estimate exposure time for each patient, in which the different conditions (Chimeric, Chimeric-Gap, Chimeric-Connected) were presented for equal number of times and in random order (but using different pictorial stimuli from the formal experiments), and exposure time was adjusted with a modified staircase procedure to achieve an error rate less than 60% in the Chimeric-Gap condition. The formal experiment used the exposure time determined for each patient. The trials in which the patients reported the right-sided component incorrectly for reasons other than neglect were scored as ineffective responses (e.g., by misnaming). In other words, the responses to the left-sided figures were analyzed only when the right-sided component had been recognized correctly. Omissions, misnamings or ambiguous answers were all scored as errors. Experiment 2 was conducted two days after Experiment 1.

### Experiment 1: Connection by a line segment

**Figure 2:**
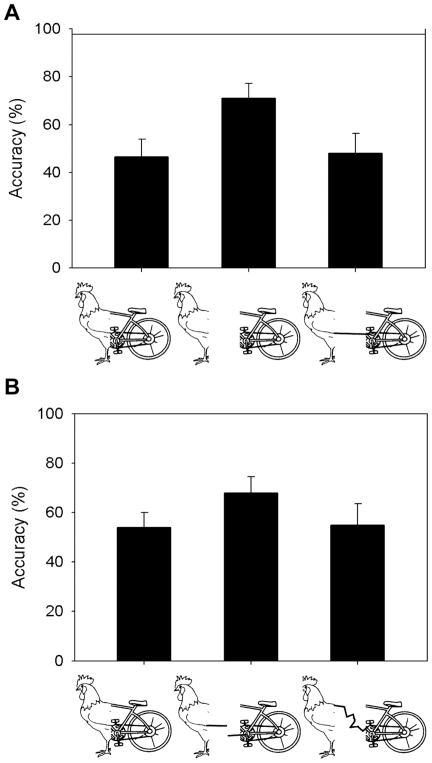
Proportion of correct identifications of the left side of the figures under the conditions of Chimeric, Chimeric-Gap, and Chimeric-Connected. (A) Experiment 1, spatial separation for Chimeric-Gap condition and connection by a line segment for Chimeric-Connected condition. (B) Experiment 2, a broken line in the gap for Chimeric-Gap condition and connection by a random curve for Chimeric-Connected condition. The error bars show the stand errors of the means.

The right half-figures under the three experimental conditions (Chimeric, Chimeric-Gap, and Chimeric-Connected) were well recognized by all patients with mean accuracy of 99.7%. The Wilcoxon signed-rank test was used to analyze the data. Figure 2A showed the accuracy of naming of the left side of the stimuli. Performance under the Chimeric-Gap condition (mean = 71%) was better than Chimeric condition (mean = 46.5%) (z = −2.68, *p* = 0.007) and Chimeric-Connected condition (mean = 48%) (z = −2.61, *p* = 0.009). There was no difference in performance between the Chimeric and Chimeric-Connected conditions (z = −0.78, *p* = 0.44).

### Experiment 2: Connection by a random curve

There were two aims to Experiment 2. First, we wished to generalize the connectedness effect by using a random curve instead of a straight line segment. Second, we controlled for the presence of the line in the connected condition by including a broken line in the gap condition. This was to exclude the possibility that the line itself rather than connectedness per se influenced the performance of the neglect patients.

## Results

The right sides of the chimeric stimuli were correctly reported by all the patients. All naming errors were made to the left sides of the chimerics. As shown in Fig. 2B, similar results were obtained. The mean accuracy of reporting the left side item with the Chimeric-Gap figures (71%) was much higher than that with the Chimeric (54%) (z = −2.67, *p* = 0.007) and Chimeric-Connected figures (53%) (z = −2.38, *p* = 0.017). There was no significant difference between the Chimeric and Chimeric-Connected conditions (z = −0.36, *p* = 0.72).

## Discussion

We have demonstrated here a negative influence of connectedness on the ability to name the left-side item of a chimeric pair. We have shown that when a central gap was introduced between the two halves of chimeric figures, the patients' ability to recognize the left sides of these figures was improved in line with prior findings [13], [21]. However, when the separated chimeric halves were connected by a straight or a randomly curved line, identification performance of the left half fell to that of ordinary chimeric figures (Experiments 1 and 2). We have also shown that the connectedness effect disappeared when the connecting line was replaced with a broken line (Experiment 2). These findings demonstrate a significant role for connectedness in how the two halves of chimetic figures are represented.

Beneficial effects of connectedness have been reported previously, but typically relate to studies employing simple geometric stimuli [11], [23]–[24]. More complex and meaningful stimuli were used in the experiments described here. The negative effects of connectedness observed may have resulted from difficulties in disengaging attention from the ipsilesional part when sufficient information is available for a response (i.e., the identity of the ipsilesional item).

Our results are relevant to the issue of whether neglect operates in space- or object-based coordinates. Egocentric neglect is associated with more anterior lesions than allocentric neglect; however, many neglect patients show both forms (with lesions associated with the temporo-parietal junction and fusiform gyrus) [20]. The better performance of the Chimeric-Gap figures relative to the Chimeric figures may be attributed to spatial effect, object effect or their combined effect. However, the different scores between Chimeric-Connected and Chimeric-Gap conditions cannot be accounted for simply from the perspective of space-based neglect. The data need to be explained in the light of an object-based reference frame. Young et al. [13] have stated that the key factor in identifying the left side of a chimeric figure is the extent to which the two halves approximates something the visual system would treat as a single object. The more like a single object, the more severe the neglect is.

Connected chimeric halves appear to be processed as an integrated whole object rather than two individual objects given the performance under Chimeric-Connected condition (mean = 50.5%) was more similar to that under Chimeric (mean = 50.25%) rather than Chimeric-Gap conditions (mean = 70.6%). According to Grabowecky *et al.* [36], an object may be a fully described and identified unit, or it may simply be a potential object based on early, preattentive visual processes such as figure-ground segregation and perceptual grouping. Palmer and Rock [37] have proposed that uniformly connected regions tend to be perceived initially as single units, namely perceptual objects. Chen [38]–[39], in his topological theory of visual perception, has also proposed a pivotal role for connectedness (as a topological property) in the establishment of object representations.

Connectedness has been adopted by researchers to dissociate the object vision and space vision. Tipper and Behrmann [40] have combined connectedness and rotation to study object effect. Two circles, one colored blue and the other red, were presented unconnected or connected by a line, and these stimuli were static or remained stationary for 1 s then underwent a 180° rotation. In the static condition reaction times for the connected stimuli were greater than those for the disconnected stimuli, which accorded with the present results of connectedness bringing about lower accuracy; It was also the case that the effects of rotation was observed for the single object (connected condition) not for the 2-object displays.

In conclusion, the present study provides a clear demonstration of a modulation effect of connectedness in neglect, and suggests that neglect could be based on the formation of perceptual units at the early stages of visual processing when connectedness plays a key role.

## References

[1] Stone SP, Halligan PW, Greenwood RJ (1993). The incidence of neglect phenomena and related disorders in patients with an acute right or left hemisphere stroke.. Age and Ageing.

[2] Vallar G, Perani D (1986). The anatomy of unilateral neglect after right-hemisphere stroke lesions. A clinical/CT-scan correlation study in man.. Neuropsychologia.

[3] Brooks JL, Wong Y, Robertson LC (2005). Crossing the midline: reducing attentional deficits via interhemispheric interactions.. Neuropsychologia.

[4] Bartolomeo P, Chokron S (2002). Orienting of attention in left unilateral neglect.. Neuroscience and Biobehavial Review.

[5] Posner MI, Walker JA, Friedrich FJ, Rafal RD (1984). Effects of parietal lobe injury on covert orienting of visual attention.. J Neurosci.

[6] Cubelli R, Speri V (2001). Naming rotated pictures and the riddle of object-centered neglect.. Cortex.

[7] Driver J, Baylis GC, Rafal RD (1992). Preserved figure-ground segregation and symmetry perception in visual neglect.. Nature.

[8] Driver J, Halligan PW (1991). Can visual neglect operate in object-centered co-ordinates? An affirmative single case study.. Cognitive Neuropsychology.

[9] Farah MJ, Brunn JL, Wong AB, Wallace MA, Carpenter PA (1990). Frames of reference for allocating attention to space: evidence from the neglect syndrome.. Neuropsychologia.

[10] Halligan PW, Marshall JC (1994). Figural perception and parsing in visuo-spatial neglect.. Neuroreport.

[11] Pavlovskaya M, Glass I, Soroker N, Blum B, Groswasser Z (1997). Coordinate frame for pattern recognition in unilateral spatial neglect.. J Cogn Neurosci.

[12] Walker R (1995). Spatial and object-based neglect.. Neurocase.

[13] Young AW, Hellawell DJ, Welch J (1992). Neglect and visual recognition.. Brain.

[14] Driver J, Pouget A (2000). Object-centered visual neglect, or relative egocentric neglect?. J Cogn Neurosci.

[15] Costello A, Warrington EK (1987). The dissociation of visuospatial neglect and neglect dyslexia.. J Neurol Neurosurg Psychiatry.

[16] Cubelli R, Nichelli P, Bonito V, De Tanti A, Inzaghi MG (1991). Different patterns of dissociation in unilateral neglect.. Brain Cogn.

[17] Riddoch MJ, Humphreys GW, Luckhurst L, Burroughs E, Bateman A (1995). “Paradoxical neglect”: spatial representations, hemisphere-specific activation and spatial cueing.. Cognitive Neuropsychology.

[18] Humphreys GW, Riddoch MJ, Robertson I, Marshall JC (1993a). Interactive attentional systems and unilateral visual neglect.. Unilateral neglect.

[19] Heinke D, Humphreys GW (2003). Attention, spatial representation, and visual neglect: Simulating emergent attention and spatial memory in the selective attention for identification model (SAIM).. Psychol Rev.

[20] Chechlacz M, Rotshtein P, Bickerton WL, Hansen PC, Deb S, Humphreys GW (2010). Separating neural correlates of allocentric and egocentric neglect: distinct cortical sites and common white matter disconnections.. Cogn Neuropsychol.

[21] Buxbaum LJ, Coslett HB (1994). Neglect of chimeric figures: two halves are better than a whole.. Neuropsychologia.

[22] Duncan J, Humphreys GW, Ward R (1997). Competitive brain activity in visual attention.. Curr Opin Neurobiol.

[23] Gilchrist ID, Humphreys GW, Riddoch MJ (1996). Grouping and extinction: Evidence for low-level modulation of visual selection.. Cogn Neuropsychol.

[24] Mattingley JB, Davis G, Driver J (1997). Preattentive filling-in of visual surfaces in parietal extinction.. Science.

[25] Humphreys GW (1998). The representation of objects in space.. Philosophical Transactions of the Royal Society.

[26] Humphreys GW, Riddoch MJ, Meyer DE, Kornblum S (1993b). Interactions between object and space systems revealed through neuropsychology.. Attention and performance XIV.

[27] Mark VM, Kooistra CA, Heilman KM (1988). Hemispatial neglect affected by non-neglected stimuli.. Neurology.

[28] Seron X, Coyette F, Bruyer R (1989). Ipsilateral influences on contralesional processing in neglect patients.. Cognitive Neuropsychology.

[29] Humphreys GW, Riddoch MJ, Meyer DE, Kornblum S (1992). Interactions between object and space systems revealed through neuropsychology.. *Attention and Performance XIV: Synergies in experimental psychology, artificial intelligence and cognitive science*.

[30] Albert ML (1973). A simple test of visual neglect.. Neurology.

[31] Azouvi P, Samuel C, Louis-Dreyfus A, Bernati T, Bartolomeo P (2002). Sensitivity of clinical and behavioural tests of spatial neglect after right hemisphere stroke.. Journal of Neurology, J Neurol Neurosurg Psychiatry.

[32] Wilson BA, Cockburn J, Halligan PW (1987). Development of a behavioural test of visuo-spatial neglect.. Archives of Physical Medicine and Rehabilitation.

[33] Halligan P, Wilson B, Cockburn J (1990). A short screening test for visual neglect in stroke patients.. Int Disabil Stud.

[34] Sarri M, Greenwood R, Kalra L, Driver J (2009). Task-related modulation of visual neglect in cancellation tasks.. Neuropsychologia.

[35] Snodgrass JG, Vanderwart M (1980). A standardized set of 260 pictures: norms for name agreement, image agreement, familiarity, and visual complexity.. J Exp Psychol Learn.

[36] Grabowecky M, Robertson LC, Treisman A (1993). Preattentive processes guide visual search: evidence from patients with unilateral visual neglect.. J Cogn Neurosci.

[37] Palmer S, Rock I (1994). Rethinking perceptual organization: The role of uniform connectedness.. Psychonomic Bulletin & Review.

[38] Chen L (2005). Topological approach to perception organization.. Visual Cognition.

[39] Chen L (1982). Topological structure in visual perception.. Science.

[40] Tipper SP, Behrmann M (1996). Object-centered not scene-based visual neglect.. J Exp Psychol Human.

